# Saturation recovery allows T_1_ mapping in the human heart at 7T with a commercial MRI scanner

**DOI:** 10.1186/1532-429X-17-S1-W1

**Published:** 2015-02-03

**Authors:** Christopher T Rodgers, Yuehui Tao, Stefan K Piechnik, Alexander Liu, Jane M Francis, Stefan Neubauer, Matthew D Robson

**Affiliations:** 1 OCMR, RDM Cardiovascular Medicine, University of Oxford, Oxford, UK

## Background

Myocardial T_1_ mapping at 1.5T and 3T distinguishes powerfully between normal and diseased tissue with focal and diffuse pathology. We recently reported the first human myocardial T_1_s at 7T using the ShMOLLI+IE inversion recovery sequence. Yet even using a unique 7T scanner with 16kW RF output, perfect magnetization inversion was impossible. We now introduce a saturation recovery method to enable myocardial T_1_ mapping with standard commercial 7T MRI scanners.

## Methods

The saturation recovery single-shot acquisition (SASHA, Chow, 2013) sequence was modified for 7T by using: an optimised train of 4xHS8 pulses to saturate and 10 FLASH readouts with saturation delays (T_S_): non-saturated, 100, 200, 300, 400, 500, 600, 650ms, 1hb + 100ms and 1hb + 700ms in a 12 heartbeat breath-hold. Data were acquired with a Siemens 7T MRI scanner (with 8kW RF), an 8-element cardiac coil and ECG gating. Signals were fitted pixelwise to "s(T_S_) = A - B exp(-T_S_ / T_1_)".

10 healthy subjects (males, 22-45yrs, 70-84kg) were recruited according to ethics regulations. For each subject, coil tuning, B_0_- shims, B_1_-shims and the central frequency were optimised over the left ventricle. Then 7T SASHA native (i.e. non-contrast) T_1_ mapping was performed in SA and HLA views.

In three subjects, post-contrast T_1_ maps were acquired ~5min after 2 peripheral bolus injections of Dotarem with a power injector (Accutron MR, MEDTRON).

## Results

**Figure 1:**
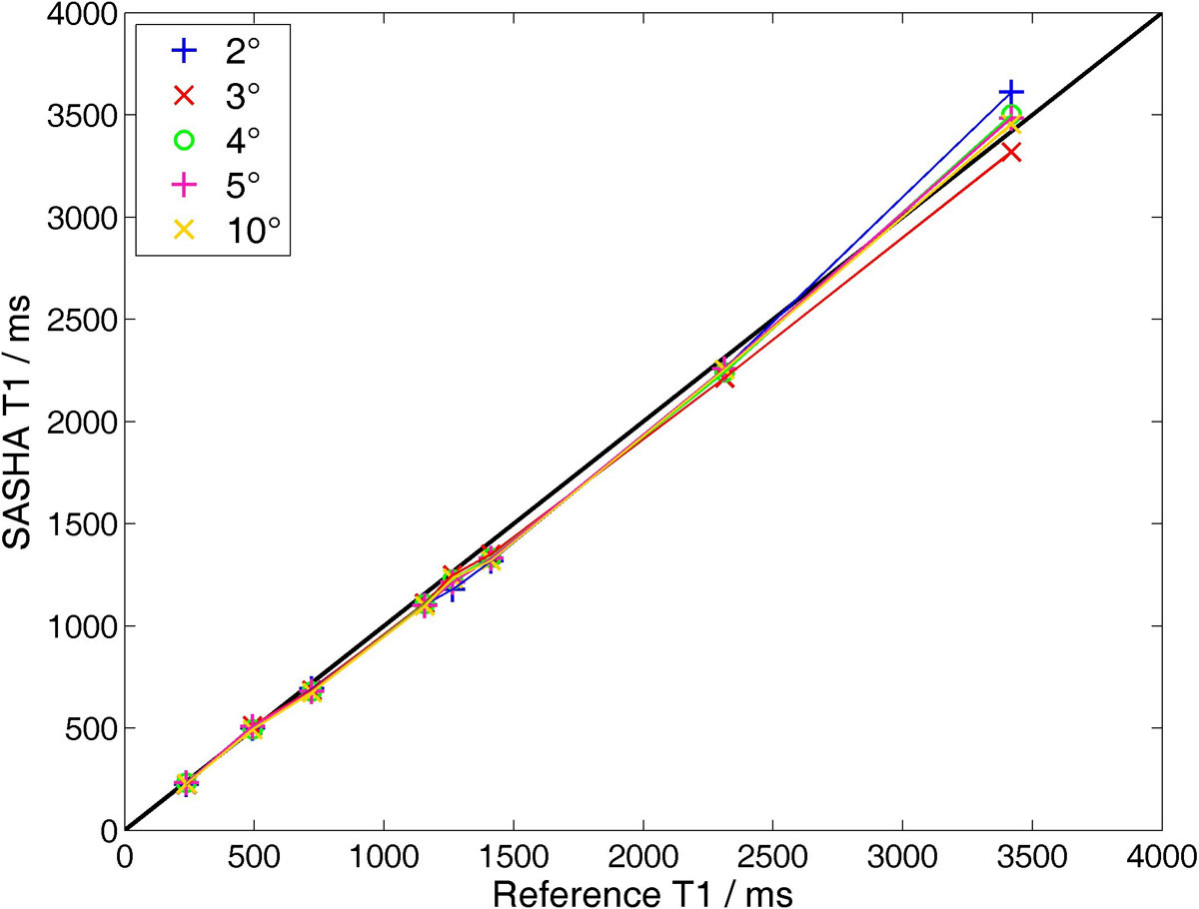
Phantom validation of 7T SASHA sequence T_1_s against inversion-recovery-spin-echo (IR-SE) reference values in a phantom comprising tubes of NiCl_2_-doped agar and carrageenan. Reference data were acquired in a 32-channel head coil (Nova Medical) to ensure sufficient B_1_^+^ for reliable inversion across the phantom. 7T SASHA data were acquired using the 8-element cardiac coil. For FLASH readout flip angles <20°, the 7T SASHA T_1_s are within 6% of the IR-SE reference T_1_s. Spin echo (SE) reference T_2_s were 50-300ms in this phantom.

"7T SASHA" T_1_s were validated against IR-SE reference T_1_s: values agreed to within 6% for readout flip angles ≤25° (Fig. 1).

In-vivo, the native 7T SASHA T_1_s in the interventricular septum were 1939±73ms. Native T_1_s in the LV blood pool showed strong artefacts, likely due to blood flow. The post-contrast T_1_s were 999, 1107 and 1674ms in myocardium and 472, 567 and 966ms in blood for Dotarem doses of 2x 50, 50 & 25 and 2x 13.5 µmol/kg in the three subjects respectively. Post-contrast T_1_ maps were acquired too soon after bolus infusion to permit calculation of extra-cellular volumes.

These myocardial T_1_s agree with our ShMOLLI+IE finding of a myocardial T_1_ = 1925 ± 48 ms. However, with ShMOLLI+IE we had to use a 4-parameter model-based fitting procedure to correct for imperfect inversion, read-out induced saturation and spin history effects. In contrast, with 7T SASHA, it is now possible to achieve comparable T_1_s using a simple 3-parameter fit (on the scanner). Note that these considerations at 7T are different to the well-known differences between MOLLI and SASHA T_1_s at 1.5 and 3T caused by imperfect inversion, T_2_ relaxation, and magnetization transfer.

## Conclusions

Saturation recovery allows T_1_ mapping in the human heart using a commercial 7T MRI scanner. T_1_s from 7T SASHA with 3-parameter fitting and ShMOLLI+IE with 4-parameter fitting are comparable in normal volunteers at 7T. Our findings hold promise for wider clinical applications of T_1_ mapping at ultra-high fields.

## Funding

Funded by the Wellcome Trust and the Royal Society [098436/Z/12/Z]; MRC; and NIHR Oxford Biomedical Research Centre.

**Figure 2:**
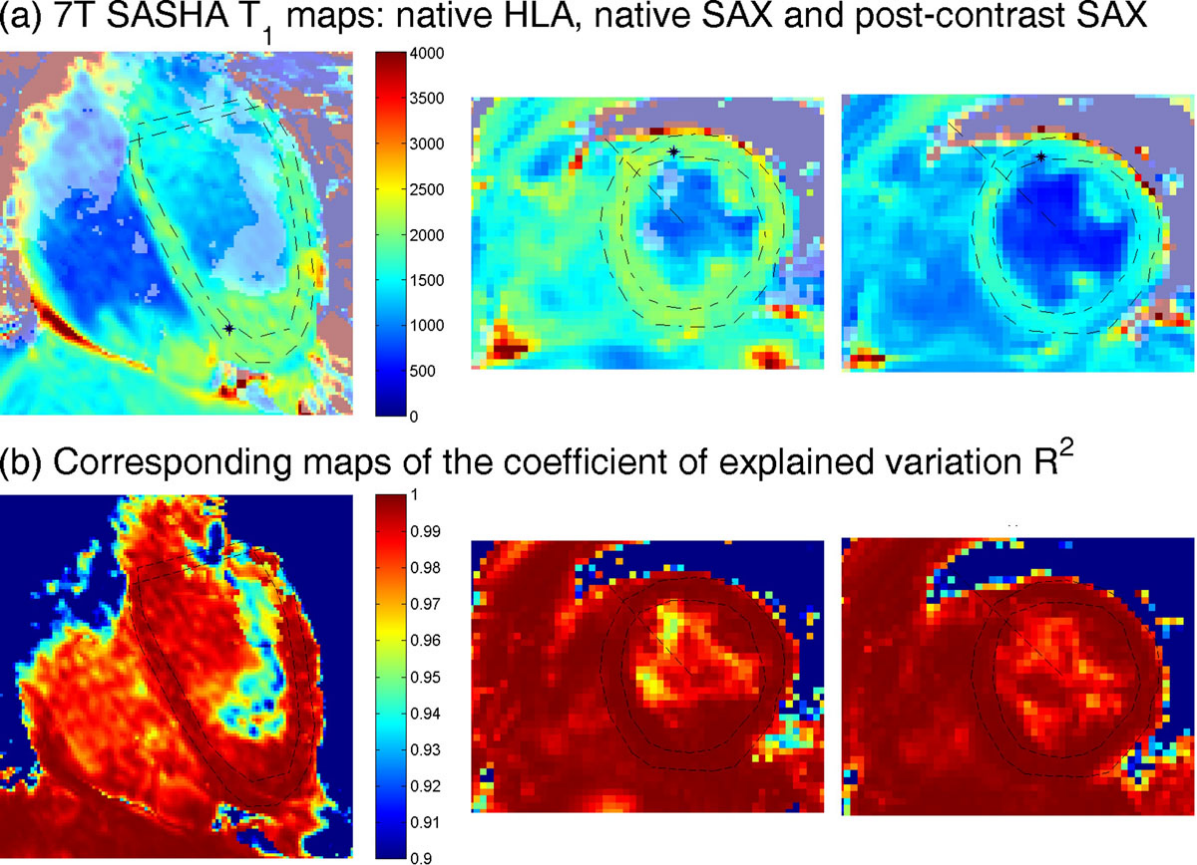
Example of in-vivo results from a healthy volunteer. Top: Myocardial T_1_ maps in a horizontal long axis view (left); mid-short-axis view (centre); and the same mid-short-axis view after administration of Dotarem Gadolinium contrast agent. Bottom: Corresponding maps of the coefficient of variation R^2^. Note how the centre of the LV blood pool has poor R^2^ and anomalous T_1_s. We believe this is due to blood flowing to/from regions of lower B_1_^+^ than the heart.

